# Inter and intralaminar excitation of parvalbumin interneurons in mouse barrel cortex

**DOI:** 10.1371/journal.pone.0289901

**Published:** 2024-06-13

**Authors:** Katherine S. Scheuer, Anna M. Jansson, Xinyu Zhao, Meyer B. Jackson

**Affiliations:** 1 Cellular and Molecular Biology PhD Program, University of Wisconsin-Madison, Madison, Wisconsin, United States of America; 2 Department of Neuroscience, University of Wisconsin-Madison, Madison, Wisconsin, United States of America; 3 Waisman Center, University of Wisconsin-Madison, Madison, Wisconsin, United States of America; Georgia State University, UNITED STATES

## Abstract

Parvalbumin (PV) interneurons are inhibitory fast-spiking cells with essential roles in directing the flow of information through cortical circuits. These neurons set the balance between excitation and inhibition and control rhythmic activity. PV interneurons differ between cortical layers in their morphology, circuitry, and function, but how their electrophysiological properties vary has received little attention. Here we investigate responses of PV interneurons in different layers of primary somatosensory barrel cortex (BC) to different excitatory inputs. With the genetically-encoded hybrid voltage sensor, hVOS, we recorded voltage changes in many L2/3 and L4 PV interneurons simultaneously, with stimulation applied to either L2/3 or L4. A semi-automated procedure was developed to identify small regions of interest corresponding to single responsive PV interneurons. Amplitude, half-width, and rise-time were greater for PV interneurons residing in L2/3 compared to L4. Stimulation in L2/3 elicited responses in both L2/3 and L4 with longer latency compared to stimulation in L4. These differences in latency between layers could influence their windows for temporal integration. Thus, PV interneurons in different cortical layers of BC respond in a layer specific and input specific manner, and these differences have potential roles in cortical computations.

## Introduction

Parvalbumin (PV) interneurons are inhibitory neurons defined by their expression of the calcium-binding protein PV [1]. These fast-spiking cells are present in cortical layers 2–6 and play critical roles in controlling excitation/inhibition balance [2, 3], sensory tuning [4, 5], gain modulation [6, 7], synchrony and timing [8], and sensory plasticity [9, 10]. PV interneurons also play a critical role in the generation of gamma oscillations [11–13], 30–80 Hz brain waves implicated in many functions including working memory, attention, and perceptual binding [14, 15]. PV interneurons and gamma oscillations have both been linked to a variety of psychiatric conditions [14, 16–18].

Primary somatosensory barrel cortex (BC) is an attractive place to study PV interneurons because of its well-defined functions and architecture [19–22]. BC is defined by the presence of barrels, cytoarchitectural units in L4 which each correspond to a single vibrissa [23]. Distinct molecular, morphological, and electrophysiological cell types form complex circuits both within and between cortical layers in BC, and PV interneurons receive dense excitatory innervation, more than 90% of which is local [24]. Two major PV interneuron morphological subgroups, basket cells and chandelier cells, are distributed differently across cortical layers in BC. Chandelier cells form axoaxonic contacts and are not present in L4 [25], while basket cells provide perisomatic inhibition and can be found in L2-6 [19, 26, 27]. These two morphological subgroups form distinct interlaminar circuits [28]. In addition to these morphological differences, PV interneurons in different layers may have different roles in functions such as intracortical and thalamic integration [19]. Optogenetically generated gamma oscillations within a given cortical layer inhibit locally within that layer but facilitate in other layers. Furthermore, peak gamma oscillation power was higher for L6 compared to L2/3 [29]. These results raise the important question of whether the roles of PV interneurons in different layers reflect differences in their responses to excitatory synaptic inputs. However a simultaneous assessment of voltage responses of PV neurons in different cortical layers has not been carried out.

Here we use the genetically-encoded hybrid voltage sensor (hVOS) to record excitatory post-synaptic potentials (EPSPs) optically from L2/3 and L4 PV interneurons in slices of mouse BC [30–32]. We determined PV interneuron response amplitude, half-width, latency, rise-time, and decay-time elicited by stimulation in L2/3 and L4. Regardless of stimulation layer, L2/3 PV interneuron responses had higher amplitudes, longer rise-times, and broader half-widths than L4 PV interneurons. Additionally, responses to stimulation in L2/3 had longer latencies than responses to L4 stimulation, even after accounting for the effect of conduction distance. By contrast, responses in these layers had similar decay-times. Thus, hVOS imaging reveals variations in electrophysiological properties of PV interneurons between cortical layers. These differences have implications for how the cortex performs computations and integrates inputs between different layers.

## Methods

### Animals

Our experiments made use of 7 mice (3 female, 4 male). Female PV-Cre driver mice (B6.129P2-Pvalb^tm1(cre)Arbr^/J, JAX strain 017320) were crossed with male Ai35-hVOS1.5 Cre-reporter mice (C57BL/6-*Gt(ROSA)26Sor*^*tm1(CAG-hVOS1*.*5)Mbja*^/J, JAX strain 031102) to generate heterozygous animals with hVOS probe targeted to PV interneurons [32]. Animal procedures were approved by the University of Wisconsin-Madison School of Medicine and Public Health Animal Care and Use Committee (IACUC protocol: M005952).

### hVOS probe

An hVOS probe was used to image voltage changes in PV interneurons. The probe used here is comprised of cerulean fluorescent protein (CeFP) tethered to the inner leaflet of the cell membrane with a truncated h-ras motif [31]. Cells expressing this probe fluoresce, and the fluorescence is modulated by Förster resonance energy transfer with dipicrylamine (DPA), a small, hydrophobic anion which partitions into the cell membrane and moves when the membrane potential changes. Depolarization drives DPA towards the CeFP and fluorescence is quenched. Repolarization drives DPA back away from the CeFP so fluorescence increases. Fluorescence thus reports voltage changes of cells expressing the hVOS probe [30, 31]. The response time of hVOS imaging was shown to be <0.5 msec due to the rapid movement of DPA through the membrane [30, 33]. As a result hVOS imaging tracks action potentials with high temporal resolution [34], and permits the determination of action potential half-widths characteristic of PV interneurons, as well as neurons targeted with Calb2, CalCrl, GAD2, and Nestin Cre-drivers [32]. Our crossing of hVOS Cre-reporter animals with PV Cre-driver animals produces mice previously shown to have 99.2% targeting specificity and to express the hVOS probe in 83% of PV interneurons [32]. Immunohistochemistry was performed in parallel with the current study using crosses of PV-Cre with the Ai14 reporter mouse (JAX 007914). Colocalization of PV with tdTomato (the fluorescent protein expressed by the Ai14 line) confirmed that recombination is specific to PV interneurons [35].

### Slice preparation

Mice 7–8 weeks old were deeply anesthetized with isoflurane and sacrificed with cervical dislocation (institutional protocol noted above). Brains were rapidly dissected and placed into ice-cold cutting solution (in mM: 10 glucose, 125 NaCl, 4 KCl, 1.25 NaH_2_PO_4_, 26 NaHCO_3_, 6 MgSO_4_, 1 CaCl_2_) bubbled with a mixture of 95% O_2_ / 5% CO_2_. After approximately five minutes, brains were mounted and cut into 300 μm thick coronal slices with a with a Leica VT1200S vibratome. Slices were placed into a chamber filled with 95% O_2_ / 5% CO_2_-bubbled artificial cerebrospinal fluid (ACSF) with the same composition as cutting solution except with 1.3 mM MgSO_4_ and 2.5 mM CaCl_2_, and allowed to recover for at least 45 minutes.

### Imaging

Imaging experiments were performed in 95% O_2_ / 5% CO_2_-bubbled ACSF containing 4 μM DPA. Slices were placed into a custom recording chamber, and viewed with a BX51 Olympus microscope. Stimulus pulses 200 μA, 180 μsec were applied with a stimulus isolator (World Precision Instruments, Sarasota, Florida) through fire-polished, ACSF-filled KG-33 glass electrodes (King Precision Glass, Claremont, California) with tip diameters of about 6–8 μm. Stimulating electrodes were positioned in L2/3 or L4 of BC using a micromanipulator. Slices were illuminated with an LED with peak emission at 435 nm (Prizmatix, Holon, Israel) through a CFP filter cube. PV interneuron responses were acquired with a CCD-SMQ camera (RedShirt Imaging, Decatur, Georgia) at 2000 Hz with 80x80 spatial resolution. Bandpass filters of 5 or 10 nm centered at 435 nm were added to the excitation pathway when resting light intensities saturated the CCD-SMQ camera. Gradient contrast and higher resolution fluorescence images were captured by directing light to a Kiralux CMOS camera (Thorlabs, Newton, New Jersey). Data acquisition and analysis was performed with custom software [36].

### Identifying individual responsive PV interneurons

PV interneurons have extensive dendritic arbors which allow them to receive input from many cells, and extensive axonal arbors to provide widespread inhibition [37–40]. Cortical PV interneurons contact about 43–50% or more of the pyramidal cells within about < 200 μm [39, 41], and fast-spiking interneuron to excitatory cell connectivity in BC can be as high as 67% for a sub-group of PV interneurons in L4 [42]. Given the dense dendritic and axonal arbors of PV interneurons, a plasma membrane label such as the hVOS probe produces broad diffuse fluorescence throughout a slice that can obscure the fluorescence from PV interneuron cell bodies. This makes it difficult to identify individual PV interneuron cell bodies.

**Fig 1:**
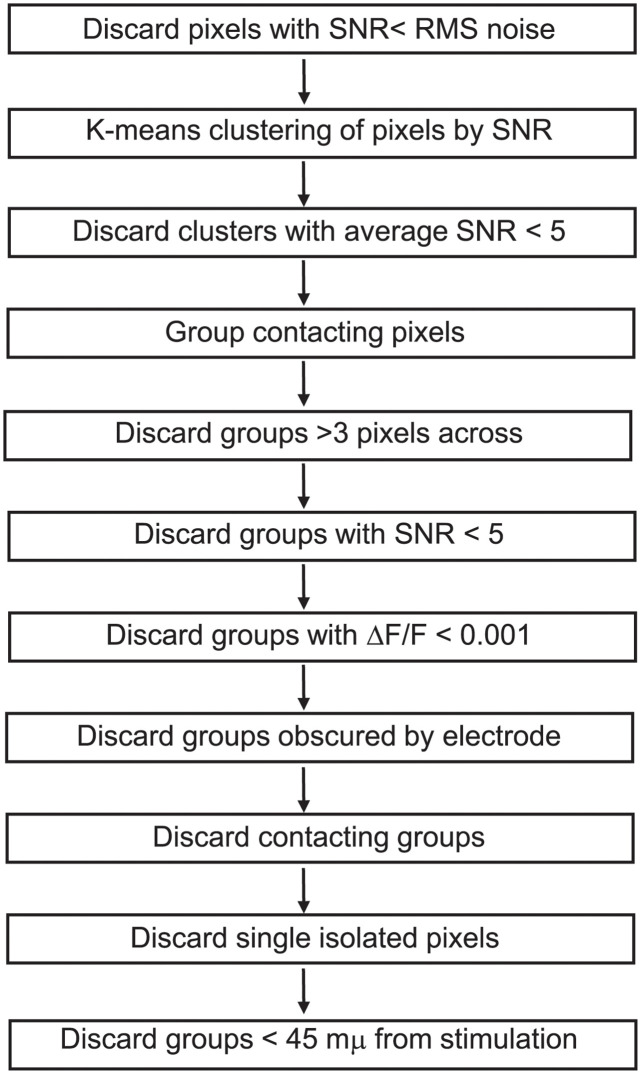
A flowchart illustrates the steps of analysis used to identify responsive neurons in maps of activity (ΔF/F) and signal-to-noise ratio (Fig 2). The sequence of steps illustrates the multiple criteria employed in identifying ROIs as single PV interneurons for analysis.

**Fig 2:**
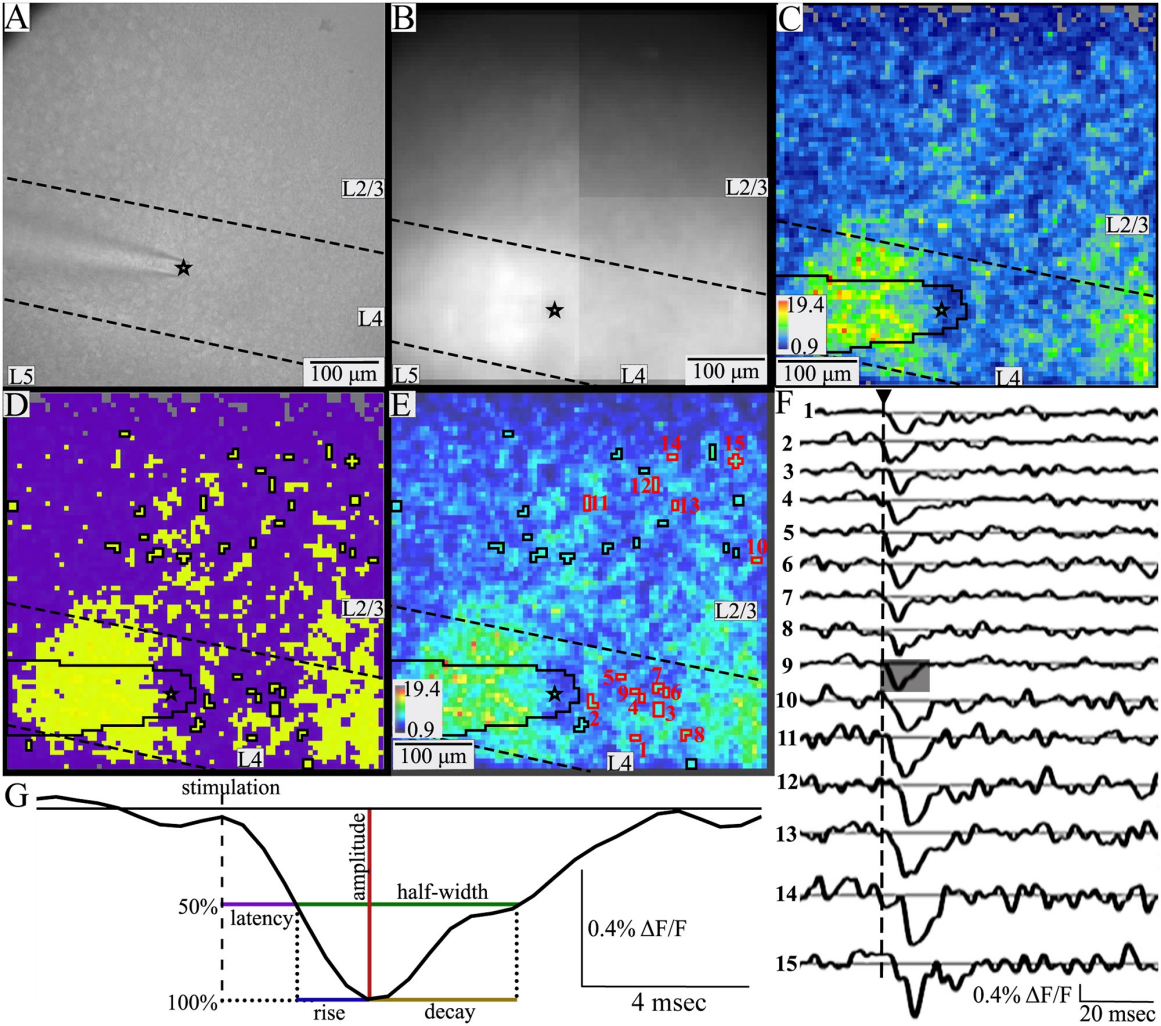
Identifying individual responsive PV interneurons. **A**. Gradient contrast image of a BC slice (with Kiralux camera). **B**. Fluorescence image of the same slice (with the CCD-SMQ camera). Black star indicates the tip of the stimulating electrode, and dashed lines indicate layer boundaries in A-E. The electrode is visible in A and B and is outlined in black in C-E. **C**. SNR heatmap of evoked responses from the same slice, with SNR coded as color according to the scale lower left. A few pixels near the top have signals below the baseline noise and were excluded from analysis (grey). **D**. K-means cluster map using the method outlined in Fig 1, based on pixels with SNR > baseline (other than gray in C). In this example the data were best fitted with two clusters with averages of 4.8 and 9.2. The yellow higher SNR cluster contains responsive PV interneurons, while the purple lower SNR cluster contains processes and unresponsive neurons. ROIs are outlined in black. **E**. An SNR heatmap from the same experiment generated by different software overlaid with identified responsive PV interneurons (33 ROIs containing 2–7 pixels, average 3.18). ROIs are outlined in black or red, with red numbers to indicate traces in F. **F**. Traces of fluorescence versus time for the PV interneurons outlined and numbered in red in E show clear depolarization in response to stimulation (triangle above and dashed vertical line). **G**. A segment of 20 msec from trace 9 in F (shaded) is expanded to illustrate response parameters analyzed here. Amplitude (red) is the maximum change in fluorescence; latency (purple) is the time from stimulation to half-maximal change; half-width (green) is the time between half-maximal changes from depolarization to repolarization; rise-time (blue) is the time between half-maximal and maximal change; decay-time (olive) is the time from peak to half-maximal fluorescence.

To address this problem, we developed a semi-automated method of analyzing imaging data for objective, reproducible identification of responsive PV interneuron somata (Fig 1). This method relied on a hybrid approach using both geometric constraints and K-means clustering of signal-to-noise ratio (SNR) values. SNR was calculated as the peak stimulus-evoked fluorescence change divided by the baseline root-mean-square fluorescence in a 20-msec pre-stimulus time window. A few pixels with a SNR below the baseline noise were discarded as clearly unresponsive (gray pixels near top, Fig 2C). For the geometric constraints, we required that a region of interest (ROI) representing a responsive PV interneuron be spatially distinct, with no shared faces. Acceptable ROIs were groups of up to nine contiguous pixels, or up to three pixels across. With 6 μm pixels this constrains groups to the size of a murine PV interneuron soma, which is approximately 15–20 μm in diameter [43–45]. This criterion excluded some larger groups of pixels as potentially representing more than one cell even though they had very clear responses with a high SNR. For example, the red-orange-yellow regions distributed through L4 and part of L2/3 in Fig 2C and 2E contained high SNR patches that were much larger than 3 pixels across and clearly contained multiple responsive cells. Pixels in areas such as these were excluded from analysis. Because a single 6 μm pixel is too small to be a cell body and could contain overlapping PV interneuron dendrites (<0.5–3 μm in rats, smaller in mice [46, 47]) and/or axons (< 1 μm, [48]), single isolated pixels were not considered to be responsive PV interneurons, again despite their high SNR. Responses from pixels obscured by the stimulating electrode (Fig 2C–2E, black outline) were also excluded. Finally, responses < 45 μm from the tip of the stimulating electrode were assumed to be the result of direct stimulation and excluded. These steps are displayed as a flowchart (Fig 1). ROIs that meet these criteria are outlined in black (Fig 2D) and black and red (Fig 2E). Red numbers over the red outlined ROIs refer to the traces in Fig 2F.

To refine this procedure, ROIs corresponding to putative responsive PV interneurons satisfying the geometric constraints were subjected to one-dimensional K-means clustering of SNR values (Fig 1). This clustering was performed on pixels with SNR above baseline noise. This served two main purposes. First, it divided pixels into groups with similar SNR values. We assume that pixel clusters with higher average SNR are more likely to contain cell bodies (yellow, Fig 2D) while those with lower average SNR are more likely to contain small processes or lack responsive cells (purple, Fig 2D). We therefore set a cutoff and clusters with an average SNR < 5 (purple, Fig 2D), as they likely contain processes or unresponsive cells. For acceptable clusters (average SNR > 5) we assumed that if pixels within a contiguous group satisfying the above-described geometric constraints have SNR values in the same K-means cluster, they are likely to represent the same cell body. K-means clustering identified groups of geometrically associated pixels with similar SNR and assigned them to specific cells. This method basically compared each pixel to its neighbors and grouped them into ROIs based on the likelihood they represent the same cell. Additionally, we discarded ROIs with average ΔF/F < 0.1% or SNR < 5. This method was conservative, implementing multiple exclusion criteria to focus on small groups of contiguous pixels with similar SNR that represent distinct, spatially separated neurons.

Traces of fluorescence versus time averaged over the pixels in ROIs identified in this way reveal depolarizations as downward deflections reflecting the voltage-dependent movement of DPA toward the fluorescent protein to quench fluorescence (Fig 2F). All traces were visually inspected to verify appropriate responses to stimulation. Responses with amplitudes more than 3 times the standard deviation above the mean value (> 1.165%) were also excluded. Such instances were very rare (4 PV interneurons total), and occurred in particularly dark areas or corners of the field of view where resting light was very low and dividing resulted in implausibly high values. Because our analysis compares PV interneuron properties based on cortical layer, occasional cells on a border between cortical layers were also excluded.

**Fig 3:**
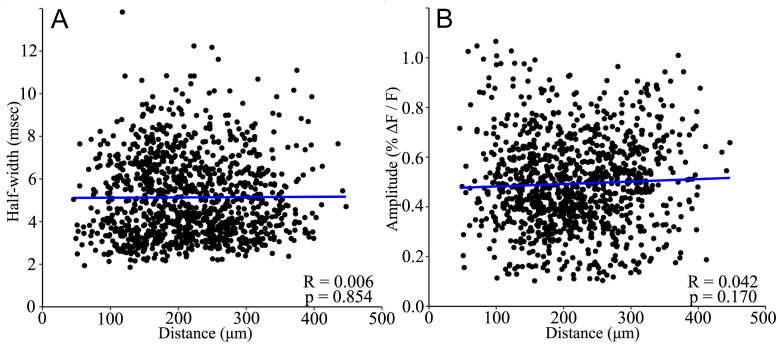
PV interneuron response half-width and amplitude do not vary with distance. **A**. Half-width was uncorrelated with distance from stimulating electrode (R = 0.006, p = 0.854). **B**. Amplitude was uncorrelated with distance (R = 0.042, p = 0.170; Pearson’s product-moment correlation for both A and B). These results support the interpretation of single-cell responses, as half-width would be expected to increase, and amplitude would decrease with distance for population responses. Each point corresponds to one ROI identified by the procedure described in Methods. Linear regression best fit lines are shown in blue. N = 1086 cells from 52 slices.

Despite the conservative nature of this analysis, our procedure identified an average of 21 responsive PV interneurons per slice. Although anatomical estimates of PV interneuron density vary widely, conservative estimates suggest that our 480x480 μm field of view may contain up to approximately 75 PV interneurons [49]. Our numbers are generally well below this, supporting our procedure as a conservative method of identifying responsive somata. This process served as a reproducible, objective, and robust method of identifying individual responsive PV interneurons. A test of validity is presented in Results (Fig 3).

Response parameters were extracted from traces of fluorescence versus time averaged over each ROI identified as representing a responsive PV interneuron (Fig 2G). Amplitude is the maximum change in fluorescence. Latency is the time from stimulation to half-maximal change in fluorescence during depolarization. To account for the effect of distance on latency, we also divided the latency by distance to the stimulating electrode (distance-normalized latency). Half-width is the time between half-maximal change in fluorescence during depolarization and repolarization. Rise-time is the time from half-maximal to peak fluorescence during depolarization. Decay-time is the time from peak to half-maximal fluorescence during repolarization. All responses were examined visually and 4 were excluded because noise resulted in anomalous start times with obvious errors in parameters.

### Data processing and statistical tests

Fluorescence traces were processed with a nine-point binomial temporal filter and a spatial filter with σ = 1. A baseline determined from a polynomial fit was subtracted. Peak fluorescence change was divided by resting light intensity to give ΔF/F. Our method of responsive cell identification yielded 1086 PV interneurons from 38 slices from 7 animals (3 female, 4 male). Relationships between distance and half-width or amplitude were evaluated with Pearson’s product-moment correlation tests using individual PV interneurons as the unit of analysis. For remaining statistical tests, values were averaged over the ROIs within each layer (excluding layers with < 8 ROIs), and this average was used as the unit of analysis.

Normality was evaluated using Shapiro-Wilks tests. All parameters were normally distributed (amplitude: W = 0.982, p = 0.591; half-width: W = 0.986, p = 0.757; rise-time: W = 0.975, p = 0.300) or log-normally distributed (distance-normalized latency: W = 0.971, p = 0.210; decay-time: W = 0.986, p = 0.750). Variance between analysis groups was evaluated with Levene’s tests. Variance did not differ significantly for any parameter based on sex (amplitude: F(1,53) = 0.903, p = 0.346; half-width: F(1,53) = 0.078, p = 0.782; distance-normalized latency: F(1,53) = 3.808, p = 0.056; rise-time: F(1,53) = 0.353, p = 0.555; decay-time: F(1,53) = 0.252, p = 0.618). The effect of sex was evaluated with *t*-tests and showed no significant impact (amplitude: t(52.857) = 0.488, p = 0.628; half-width: t(50.648) = -1.018, p = 0.314; distance-normalized latency: t(52.438) = -1.252, p = 0.216; rise-time: t(48.567) = -0.652, p = 0.517; decay-time: t(52.005) = -0.893, p = 0.376).

Variance did not differ significantly based on PV interneuron layer or stimulation layer for any parameter (amplitude (F(3,51) = 0.465, p = 0.708); half-width (F(3,51) = 0.593, p = 0.623); distance-normalized latency (F(3,51) = 1.052, p = 0.378); rise-time (F(3,51) = 0.596 p = 0.621); decay-time (F(3,51) = 0.223, p = 0.880)). The effects of stimulation layer and/or PV interneuron residence layer on each parameter were therefore evaluated with ANOVA and post-hoc Tukey’s honestly significant differences tests.

### Code availability

R code, Python code, and custom software available on request.

## Results

### Validation of single cell identification

We tested our procedure of cell identification by examining variations with distance. If pixel groups actually represent multiple neighboring neurons rather than a single neuron, we would expect response half-width to broaden and response amplitude to decrease with distance from the stimulating electrode, as the activation of more distant groups should be less synchronous compared to closer groups. In plots versus distance neither parameter was significantly correlated with distance (Fig 3, half-width: R = 0.006, p = 0.854; amplitude: R = 0.042, p = 0.170), indicating that ROIs do not contain more than one PV interneuron.

#### PV interneuron responses vary between cortical layers

**Fig 4:**
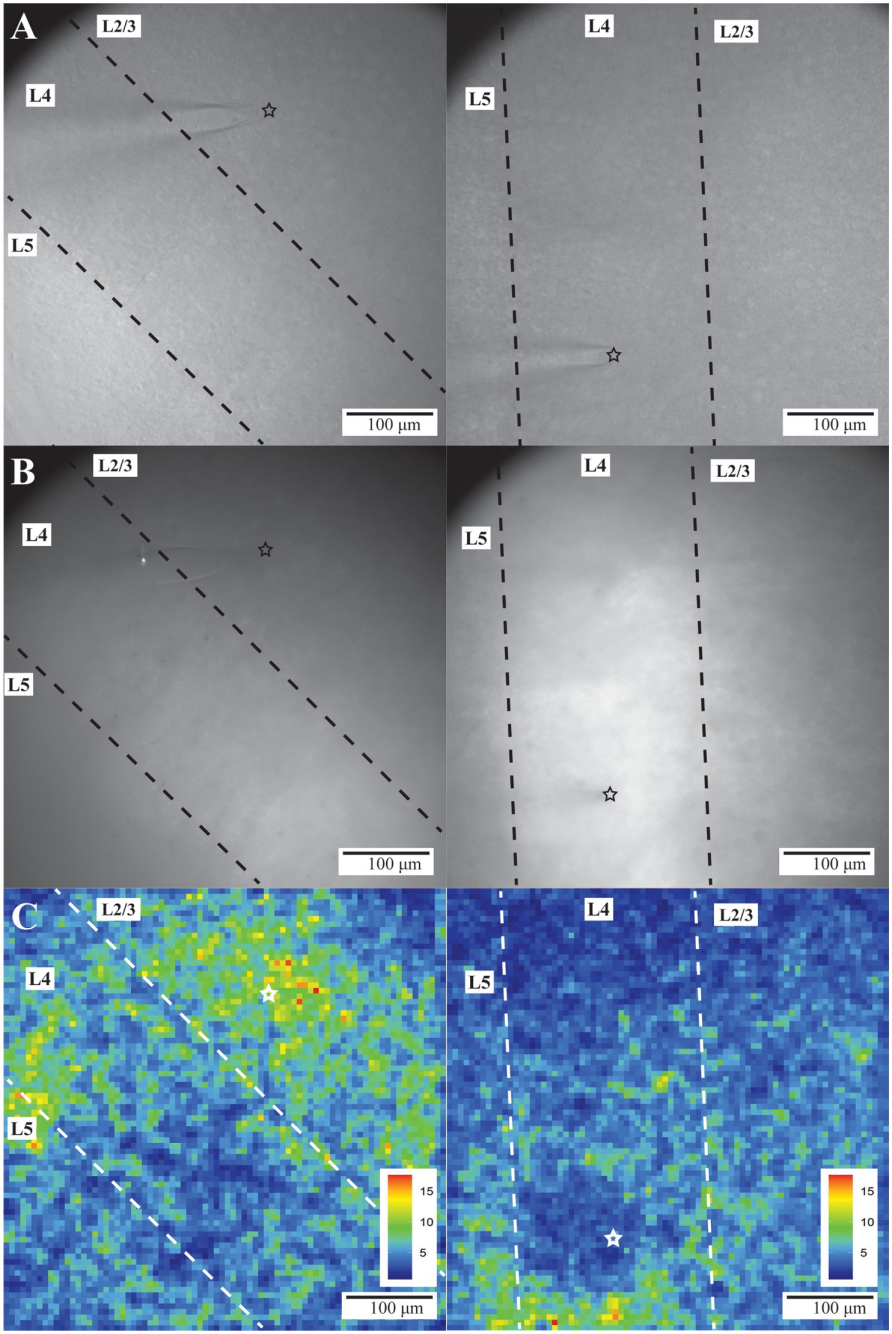
PV interneuron responses in BC. Gradient contrast (A) and fluorescence (B) images of two different slices of BC. L2/3 through L5 are visible within the fields of view. The tip of the stimulating electrode (black or white star) is visible in L2/3 (left) or L4 (right) in A-C. Dashed lines separate layers. C. SNR heatmaps of evoked responses from the slices shown in A and B. Warmer colors correspond to higher SNR and more responsive PV interneurons (color scales and ranges–lower right).

Stimulation in L2/3 (Fig 4A and 4B, left) or L4 (Fig 4A and 4B, right) elicited responses across L2/3 through L5 as shown in SNR heatmaps (Fig 4C). L2/3 and L4 PV interneuron response parameters are presented in Table 1. Comparisons are presented in bar graphs below and will be discussed in detail.

**Table 1:** PV interneuron response parameters based on residence layer and stimulation layer.

	L2/3 Stimulation		L4 Stimulation	
	L2/3 PV interneurons (N = 335, 18 slices)	L4 PV interneurons (N = 259, 15 slices)	L2/3 PV interneurons (N = 332, 13 slices)	L4 PV interneurons (N = 160, 9 slices)
Amplitude (% Δ F/F)	0.567 ± 0.010	0.363 ± 0.009	0.556 ± 0.009	0.422 ± 0.013
Half-width (msec)	5.29 ± 0.120	4.82 ± 0.100	5.45 ± 0.106	4.71 ± 0.131
Raw Latency (msec)	2.79 ± 0.105	3.89 ± 0.109	3.28 ± 0.090	2.38 ± 0.121
Distance-normalized latency (msec/μm)	0.015 ± 0.0004	0.017 ± 0.0004	0.013 ± 0.0003	0.014 ± 0.0005
Rise-Time (msec)	2.32 ± 0.085	2.17 ± 0.079	2.48 ± 0.079	2.02 ± 0.100
Decay-Time (msec)	2.97 ± 0.091	2.64 ± 0.073	2.97 ± 0.077	2.69 ± 0.097

Values are mean ± SE, N = number of neurons.

**Fig 5:**
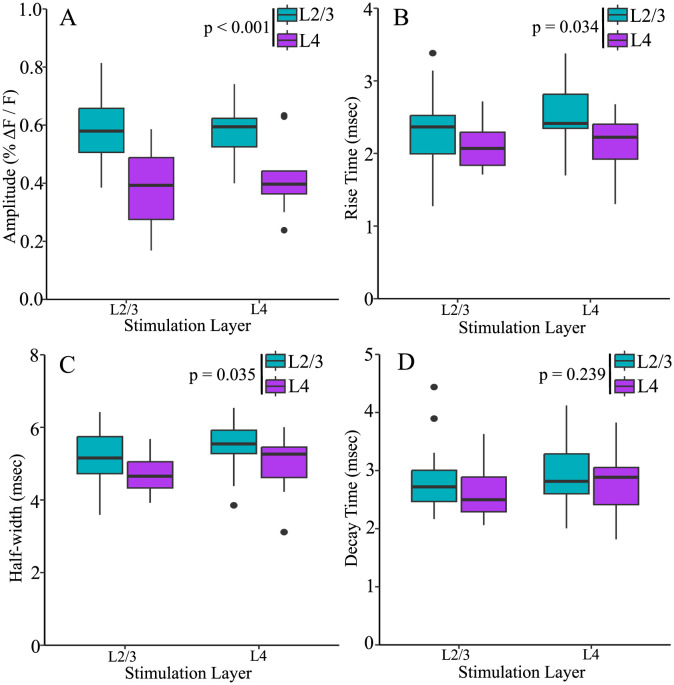
Amplitude, rise-time, and half-width of PV interneurons residing in different layers. L2/3 PV interneuron responses (blue) had higher amplitudes (**A**), longer rise-times (**B**), and broader half-widths (**C**) than responses from PV interneurons in L4 (purple). Decay-time (**D**) did not differ based on PV interneuron residence layer. Stimulation layer did not significantly impact amplitude, half-width, rise-time, or decay-time. All boxplots are nonparametric.

PV interneuron residence layer significantly impacted amplitude (Fig 5A, F(1,51) = 32.438, p < 0.001), rise-time (Fig 5B, F(1,51) = 4.753, p = 0.034), and half-width (Fig 5C, F(1,51) = 4.710, p = 0.035), but not decay-time (Fig 5D, F(1,51) = 1.422, p = 0.239). Regardless of stimulation layer, PV interneuron response amplitudes (0.579 ± 0.019, mean ± SE) were 46% larger in L2/3 than in L4 (0.396 ± 0.026, mean ± SE, p < 0.001). Rise-times for PV interneurons residing in L2/3 (2.39 ± 0.090 msec) were also longer than those in L4 (2.13 ± 0.071 msec, p = 0.034). L2/3 PV interneuron response half-widths (5.26 ± 0.144 msec) were broader than L4 response half-widths (4.82 ± 0.138 msec, p = 0.035).

**Fig 6:**
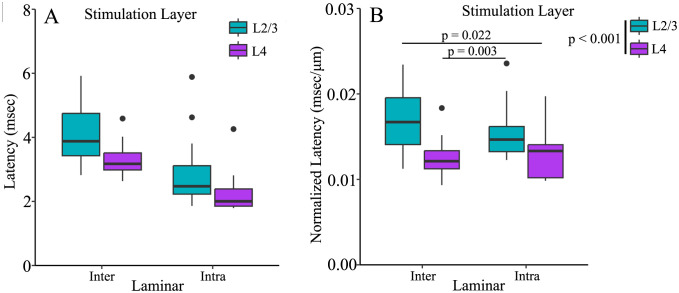
PV interneuron response latency depends on stimulation layer. **A**. Raw latencies (not divided by distance) and **B**. Normalized latencies (to distance) for PV interneuron responses to stimulation in L2/3 (orange) or L4 (green). Regardless of residence layer, responses elicited by L2/3 stimulation (orange) have longer distance-normalized latencies than those elicited by L4 stimulation (green, F(1,51) = 16.478, p < 0.001). Both boxplots are nonparametric.

Latency also differed between cortical layers (Fig 6). Unlike amplitude, rise-time, and half-width, however, latency also depended on stimulation layer. To analyze variations in latency we must take into account the influence of distance and conduction time [50]. Distances from the electrode tip to PV interneurons in L2/3 (180 ± 9.3 μm) and L4 (178 ± 10.6 μm) were similar for intralaminar stimulation, as were distances of cells in L2/3 (237 ± 6.5 μm) and L4 (260 ± 10.6 μm) for interlaminar stimulation. However, average interlaminar distance (248 ± 5.6 μm) was 39% longer than intralaminar distance (179 ± 7.02 μm). As expected, distance significantly impacted raw latency (F(1,53) = 49.62, p < 0.001). To account for this effect, we compared response latencies divided by distance from the tip of the stimulating electrode (distance-normalized latency). Stimulation layer significantly affected distance-normalized latency (F(1,51) = 16.478, p < 0.001). Regardless of PV interneuron residence layer, distance-normalized latencies of responses to stimulation in L2/3 (0.0162 ± 0.0006 msec/μm) were significantly longer than those to stimulation in L4 (0.0128 ± 0.0006 msec/μm, p < 0.001). PV interneurons residing in L2/3 responded to interlaminar L4 stimulation (0.013 ± 0.0006 msec/μm) more quickly than intralaminar L2/3 stimulation (0.016 ± 0.0007 msec/μm, t(26.992) = 3.243, p = 0.003). However, L4 PV interneurons responded to interlaminar L2/3 stimulation (0.017 ± 0.001 msec/μm) more slowly than to intralaminar L4 stimulation (0.013 ± 0.001 msec/μm, t(16.047) = 2.537, p = 0.022). Thus, stimulation layer impacts latency in a manner which cannot be attributed to differences in distance.

## Discussion

Here we used the genetically-encoded hybrid voltage sensor hVOS to investigate PV interneurons in BC. We observed depolarizing responses of L2/3 and L4 PV interneurons to stimulation of both these layers. We developed a semi-automatic method of identifying individual responsive PV interneurons that combines geometric considerations with statistical K-means clustering of SNR. This method located responsive cells within the high background fluorescence produced by the extensive arborization of PV interneurons. Based on the assumption that ROIs with high SNR correspond to cell bodies, we were able to identify cell bodies of ~20 responsive PV interneurons per slice, and determine response amplitude, half-width, latency, rise-time, and decay-time of their responses to synaptic excitation. To test our method, we plotted half-width and amplitude versus distance from the electrode tip (Fig 3). The absence of correlations suggests that our pixel clusters contain single cells rather than multiple cells. The parameters reported in this study represent basic elements of cortical circuitry that may be useful in the development of accurate computational models designed to recapitulate the function of fast-spiking interneurons in BC microcircuit computations [51], and the generation of synchronous activity [8, 52, 53].

Previous hVOS studies of PV interneuron activity in somatosensory cortex determined that spike-like responses similar to action potentials had a peak ΔF/F of 2.4% [32], while unitary synaptic responses elicited by an action potential in a single excitatory neuron ranged from 0.2–0.4% [54]. Assuming ~100 mV action potentials, the mean amplitude of 0.49% reported here can be estimated as approximately 21 mV. The responses reported here are 64% larger than unitary excitatory responses and about one fifth the amplitude of spike-like responses. They are clearly too small to be action potentials, and are likely to represent EPSPs elicited by an average of about two excitatory neurons. Consistent with our assessment that these responses are synaptic potentials, our half-widths of ~5.14 msec are 3.6 times broader than half-widths of spikes recorded from PV interneuron with hVOS [32]. Our half-widths fall in the range of other studies of subthreshold, synaptic responses of PV interneuron in murine cortex of 4.6 to 22.3 msec [51, 55–63]. Our 2.8 msec 50% decay-time corresponds to an exponential decay-time of 4.1 msec, which is within the range (3.5 to 12 msec) of previously reported values for EPSPs in murine cortical PV interneurons [64–67]. Thus, parameter values reported here fall within the range of previous reports, and reveal how PV interneuron properties vary depending on location and source of excitation.

**Fig 7:**
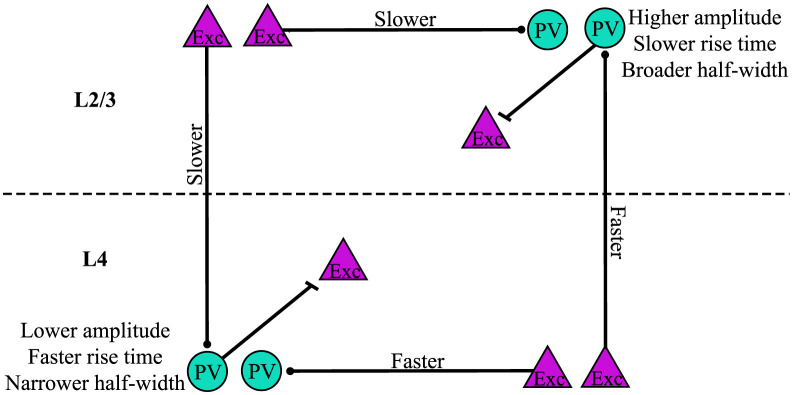
Summary of PV interneuron response differences. Amplitude, distance-normalized latency, rise-time, and half-width vary based on cortical layer. PV interneurons (teal circles) residing in L2/3 had higher amplitudes, slower rise-times, and broader half-widths compared to those in L4. Distance-normalized latencies of responses to stimulation of excitatory cells (purple triangles) in L2/3 were longer than those of responses to stimulation in L4.

Response amplitude, distance-normalized latency, rise-time, and half-width all varied, and Fig 7 illustrates the key differences. Regardless of stimulation layer, response amplitude was greater for PV interneurons in L2/3 compared to L4. This may reflect a greater role in feedback inhibition of L4 [1, 28, 68]. The different distributions of chandelier cells and basket cells may be relevant to these results. Whereas chandelier cells are distributed through most layers, basket cells reside primarily in L4 [19, 25–27]. The responses in L4 could thus arise from basket cells and the responses in L2/3 PV could arise from both chandelier and basket cells. EPSP amplitude depends on a wide variety of factors including number of inputs, dendritic location, and ion channel and receptor makeup [63, 64, 69–73]. The increased EPSP amplitude in L2/3 compared to L4 PV interneurons may therefore reflect some of these factors. PV interneuron EPSP amplitudes are more than twice as large if the presynaptic excitatory cell and PV interneuron are reciprocally connected [65]. Thus, the larger EPSP amplitudes of L2/3 PV interneurons could be related to higher reciprocal connectivity. Calcium-permeable AMPA receptors have also been shown to impact fast-spiking interneuron synaptic responses [71, 72], and differences in their distribution between layers could contribute to the present findings.

PV interneuron residence layer also impacted rise-time and half-width. L2/3 PV interneuron responses had longer rise-times and half-widths than responses of PV interneurons in L4. Differences in rise-time could reflect dendritic location, presynaptic release kinetics, or AMPA receptor subunit composition. Compared to other types of interneurons and pyramidal cells, PV interneurons in CA1 express higher levels of AMPA receptor subunit GluA1, higher levels of auxiliary proteins regulating AMPA receptors, and especially high levels of GluA4 [74]. Knockout of GluA4, but not GluA3, decreases rise-time [75]. AMPA receptors on PV interneurons can have multiple subunit combinations [76]. Therefore, differences in rise-time based on PV interneuron residence layer might reflect layer-specific variation in AMPA receptor subunit composition. Longer rise-times for PV interneurons in L2/3 compared to L4 likely contribute to the broader half-widths of L2/3 PV interneurons. These differences could in part be due to the different distributions of chandelier cells and basket cells [19, 25–27]. Longer rise-times and half-widths are likely to impact the way in which PV interneurons modulate sensory timing and synchony [4, 5, 8].

In addition to differences in amplitude, rise-time, and half-width, we also observed differences in latency between cortical layers. However, while the former properties depended on the layer in which PV interneurons resided, latency differed depending on the location of the inputs. Accounting for the effect of distance, both L2/3 and L4 PV interneuron responses to L2/3 stimulation had significantly longer latencies compared to responses to L4 stimulation. Because stimulation layer impacted latencies regardless of the layer in which the PV interneurons resided, this difference may reflect a property of the excitatory input rather than postsynaptic properties of PV interneurons. The inputs could have a faster conduction time along their axons or more direct axonal paths to their targets. It is also possible that the latency differences reflect differences in the kinetics of neurotransmitter release between axons originating in L2/3 and L4. The ability of L4 to activate PV interneurons more rapidly could provide a temporal advantage in engaging feedforward inhibition to limit the temporal window of sensory inputs [1, 28, 68].

PV interneurons in L4 respond to whisker stimulation with slightly shorter latencies than PV interneurons in L2/3, though the difference is not statistically significant [8]. Here we observed significant latency differences that reflect times for internal processing rather than primary sensory responses. The shorter latencies of L2/3 PV interneuron responses to interlaminar L4 excitation compared to the reverse pathway of L4 PV interneuron responses to interlaminar L2/3 excitation will enable feedforward excitation along the canonical route from L4 to L2/3 PV interneurons to occur more quickly than L2/3 to L4 feedback. Inhibition plays a key role in coincidence detection by controlling the temporal integration window [77] and the synchrony of excitatory neurons [8]. Because PV interneurons fire rapidly, they are particularly well-suited to tightly constraining integration within their synaptic targets. Compared to L4, which processes more basic sensory information such as touch and whisking, L2/3 functions are related to more complex somatosensory processing such as object localization [78], stimulus-specific adaptation [79], texture discrimination [80], and social touch [81]. The particularly rapid L4 to L2/3 excitation reported here could narrow the integration window set by L2/3 PV interneurons on their targets, and therefore may impact these higher-level sensory processes. Due to the limited the field of view of our optical system, we chose here to focus on L2/3 and L4. Given that L5 and L6 also contain PV positive chandelier cells, future studies in these layers may reveal additional layer-based distinctions in physiological function.

This work demonstrates the utility of hVOS voltage imaging as a technique to examine cortical circuitry of a specific type across multiple cortical layers. Imaging has the advantage of being able to record from many cells simultaneously, thus greatly increasing the amount of data obtained from a single experiment. This approach can be used to measure response parameters such as amplitude, half-width, latency, rise-time, and decay-time, which are important for computations. It also provides an opportunity to compare these response parameters across cortical layers. Here we observed layer-based differences in amplitude, rise-time, and latency which hold implications for how BC integrates interlaminar and intralaminar inputs. Whether other sensory cortices exhibit variations in interneuron behavior is an important question that will bear on how circuits adapt to different forms of sensory input. Future work building on this approach has the potential to address the circuit functions of PV interneurons as well as other specific cell types throughout the brain.

## Supporting information

S1 Checklist The ARRIVE guidelines 2.0: Author checklist. (PDF)
